# Adenosine and adenosine-5′-monophosphate ingestion ameliorates abnormal glucose metabolism in mice fed a high-fat diet

**DOI:** 10.1186/s12906-018-2367-6

**Published:** 2018-11-14

**Authors:** Ardiansyah, Yuto Inagawa, Takuya Koseki, Afifah Zahra Agista, Ikuo Ikeda, Tomoko Goto, Michio Komai, Hitoshi Shirakawa

**Affiliations:** 1 Laboratory of Nutrition, Graduate School of Agricultural Science, Tohoku University, Sendai, 980-0845 Japan; 2 Department of Food Science and Technology, Universitas Bakrie, Jakarta, 12920 Indonesia; 3 Faculty of Agriculture, Yamagata University, Tsuruoka, 997-8555 Japan; 4 Laboratory of Food and Biomolecular Science, Graduate School of Agricultural Science, Tohoku University, Sendai, 980-0845 Japan; 5 International Education and Research Center for Food Agricultural Immunology, Graduate School of Agricultural Science, Tohoku University, Sendai, 980-0845 Japan

**Keywords:** Adenosine, Adenosine-5′-monophosphate, Glucose metabolism, High-fat diet

## Abstract

**Background:**

We have previously reported that ingestion of adenosine (ADN) and adenosine-5′-monophosphate (AMP) improves abnormal glucose metabolism in the stroke-prone spontaneously hypertensive rat model of non-obesity-associated insulin resistance. In this study, we investigated the effect of ADN and AMP ingestion on glucose metabolism in mice with high-fat diet-induced obesity.

**Methods:**

Seven-week-old C57BL/6 J mice were administered distilled water (as a control), 10 mg/L ADN, or 13 mg/L AMP via their drinking water for 14 or 25 weeks, during which they were fed a high-fat diet. Oral glucose tolerance test (OGTT) was conducted on 21-week-old mice fasted for 16 h. Insulin tolerance test (ITT) was performed on 22-week-old mice fasted for 3 h. Blood and muscle were collected for further analysis of serum parameters, gene and protein expression levels, respectively.

**Results:**

Glucose metabolism in the ADN and AMP groups was significantly improved compared with the control. OGTT and ITT showed that ADN and AMP groups lower than control group. Furthermore, phosphorylation of AMP-activated protein kinase (AMPK) and mRNA levels of genes involved in lipid oxidation were enhanced in the skeletal muscle of ADN- and AMP-treated mice.

**Conclusion:**

These results indicate that ingestion of ADN or AMP induces activation of AMPK in skeletal muscle and mitigates insulin resistance in mice with high-fat diet-induced diabetes.

**Electronic supplementary material:**

The online version of this article (10.1186/s12906-018-2367-6) contains supplementary material, which is available to authorized users.

## Background

The prevalence of obesity has increased because of changing lifestyles, including dietary habits, in both developed and developing countries [1]. Hypertension, diabetes, dyslipidemia, and cardiovascular diseases associated with obesity have been recognized as diseases of worldwide importance [2, 3]. Excessive energy intake causes increased presence of non-esterified fatty acids in the blood and diacylglycerol and palmitoyl-CoA in the liver and skeletal muscle, followed by heightened oxidative stress [4, 5], factors that are known to induce insulin resistance.

Given that insulin resistance is a major pathogenic event in the occurrence and progression of lifestyle-associated diseases, its alleviation is a key strategy for the prevention of such conditions. Adenosine-5′-monophosphate (AMP)-activated protein kinase (AMPK) has been reported to attenuate insulin resistance, since it enhances lipid oxidation and glucose uptake in the liver and skeletal muscle, in which it regulates the expression of β-oxidation-associated genes. Therefore, much attention is being paid to AMPK as a potential therapeutic target in the treatment of insulin resistance [6, 7].

Adenosine (ADN) is an endogenous purine nucleoside but also derives from AMP in food via the action of nucleotidase in the small intestine, from which it is absorbed into the body. Cellular ADN levels are regulated by both efflux and influx transporters [8, 9], and this molecule participates in diverse cellular functions, including the inflammatory immune response and lipid metabolism in the liver [10–12].

Various studies have provided evidence to support the role of ADN in health. For example, studies in both rats and humans demonstrated that oral ingestion of ADN in sucrose solutions significantly decreased blood glucose and insulin levels through inhibition of α-glucosidase activity by ADN [13, 14]. ADN has the capacity to attenuate the proliferation of both human and rat glomerular mesangial cells, which are related to hypertension and diabetes [15]. Furthermore, prior research using Sprague–Dawley rats has shown that ADN attenuates high-fat diet-induced increases in blood glucose and insulin, suppresses elevation of plasma corticosterone levels, rectifies altered nutrient transporter expression profiles, and prevents upregulation of TNF-α in the intestine [16]. On the other hand, AMP is well known as a purine nucleotide and participant in ATP metabolism. It has been approved by the FDA as a food additive to block bitter taste or enhance flavor [17, 18]. In addition, the functional properties of AMP have been implicated in thermoregulation of men and induction of hypothermia through ADN receptors [19].

Our group has examined the effect of ADN and AMP administration on the stroke-prone spontaneously hypertensive rat model of non-obesity-induced hypertension and insulin resistance [20, 21]. We found that ingestion of ADN and AMP activates AMPK in skeletal muscle and ameliorates insulin resistance and impaired glucose metabolism. The aim of the present study was to investigate the influence of ADN and AMP ingestion on glucose metabolism in a mouse model of obesity-associated insulin resistance induced by a high-fat diet.

## Methods

### Animal experiments

**Fig. 1:**
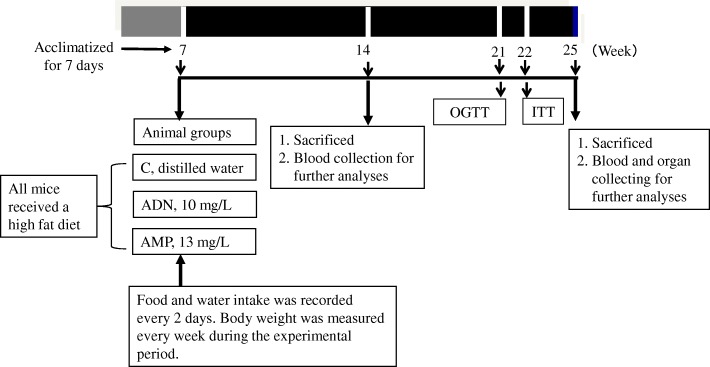
Workflow diagram of the experiments. C, control group; ADN, adenosine group; AMP, adenosine-5′-monophosphate group; OGTT, oral glucose tolerance test; ITT, insulin tolerance tests

Male C57BL/6 J mice (CLEA Japan Inc., Tokyo, Japan) were housed in polycarbonate cages (three mice per cage) under controlled conditions (temperature, 23 ± 3 °C; humidity, 50 ± 10%; 12/12 h light/dark cycle). The mice were sorted by body weight before beginning the study, and those used in experiments were of the same weight. The experimental design of the present study was approved by the Animal Research-Animal Care Committee of Tohoku University. The mouse model of diet-induced obesity has become popular as a tools for understanding high-fat in human and the development of obesity. C57BL/6 J mice as a good model for diet-induced obesity that has correlated closely with human obesity progression [22]. All experiments were conducted in accordance with the guidelines issued by this committee and Japanese legislation (2005). The mice were acclimatized for 7 days with free access to conventional non-purified diet (F-2, Funabashi Farm Co., Ltd., Chiba, Japan) and distilled water. After this period, the 7-week-old mice were administered distilled water (control), ADN, or AMP for 14 or 25 weeks. ADN (Wako Pure Chemical Industries, Osaka, Japan) and AMP (kindly provided by Yamasa Co., Chiba, Japan) were used at 10 and 13 mg/L in distilled water, respectively. All mice received a high-fat diet (HFD32, CLEA Japan Inc.). HFD32 is super-high-fat diet with 32% of crude fat and calorie rate 60% from gross energy (fat kcal %). Food and water intake was recorded every 2 days, and body weight was measured every week during the experimental period. At the end of the experiment, mice were euthanized by decapitation after 6 h of fasting. Blood was collected, and serum was immediately separated by centrifugation and stored at − 20 °C until analysis. Skeletal muscle (the quadriceps femoris muscle) was excised and kept at − 80 °C until needed. The workflow diagram of the experiment is shown in Fig. 1.

### Tolerance tests

We subjected mice in the 25-week experimental groups to both oral glucose tolerance tests (OGTT) and insulin tolerance tests (ITT). OGTT were conducted on 21-week-old mice fasted for 16 h. Blood for glucose measurement was collected from the tail vein before (0 min) and 15, 30, 60, and 120 min after administration of glucose at 2.0 g/kg body weight via a gastric tube. Blood glucose levels were measured using a StatStrip Glucose Xpress Meter (Nova Biomedical Co., Waltham, MA, USA).

ITT were performed on 22-week-old mice fasted for 3 h. Blood for glucose measurement was collected from the tail vein before (0 min) and 15, 30, and 60 min after intraperitoneal administration of insulin (Humulin R, Eli Lilly & Co., Indianapolis, IN, USA) at 0.75 U/kg body weight. Blood glucose levels were measured with a StatStrip Glucose Xpress Meter.

### Serum parameters

Serum levels of glucose, triglycerides, and non-esterified fatty acids were measured by enzymatic colorimetric methods (Wako Pure Chemical Industries). Serum insulin and adiponectin levels were tested using rat insulin (Morinaga Institute of Biological Science, Inc., Yokohama, Japan) and mouse/rat adiponectin (Otsuka Co., Tokyo, Japan) ELISA kits, respectively. Blood samples were collected, centrifuged at 1870×g for 15 min at 4 °C in a centrifuge (CF7D2; Hitachi Co. Ltd., Tokyo, Japan) and stored at − 80 °C until required for later analyses.

### RNA preparation and quantitative RT-PCR

Total RNA was isolated from muscle tissue with the guanidine isothiocyanate-based reagent Isogen (Nippon Gene, Tokyo, Japan), according to the manufacturer’s instructions. The isolated RNA was treated with RNase-free DNase (Qiagen, Hilden, Germany) for 10 min at room temperature before being purified using an RNeasy Mini kit (Qiagen). The ratio of absorbance at wavelengths of 260 and 280 nm was measured, and agarose gel electrophoresis was performed for quantitative and qualitative analysis of the isolated RNA. Four micrograms of total RNA was used as a template to synthesize cDNA. The RNA was denatured in the presence of oligo (dT), random primers, and 10 mmol/L dNTP (Amersham Biosciences, Tokyo, Japan) at 65 °C for 5 min. It was then incubated in a 20-μL volume with 50 mmol/L Tris-HCl (pH 8.3) containing 0.1 mol/L DTT, 50 U SuperScript III reverse transcriptase (Invitrogen, Carlsbad, CA, USA), and 20 U RNaseOUT RNase inhibitor (Invitrogen) at 25 °C for 5 min, 50 °C for 60 min, and 70 °C for 15 min. Aliquots of the resulting cDNA were used as templates in subsequent quantitative PCR using an Applied Biosystems (Foster City, CA, USA) 7300 Real-Time PCR System and SYBR Premix Ex Taq (Takara Bio Inc., Kusatsu, Japan) according to the manufacturers’ instructions. Relative target gene expression levels were normalized to those of eukaryotic elongation factor-1α1 mRNA [23]. The target sequences were amplified using primers specific to the corresponding cDNA (Additional file 1: Table S1).

### Western blot analysis

Skeletal muscle lysate was prepared after removal of adipose tissue by homogenizing the muscle in ice-cold phosphate-buffered saline containing inhibitors of proteinase (Complete Proteinase Inhibitor Cocktail, Roche Applied Science, Mannheim, Germany) and phosphatase (PhosSTOP Phosphatase Inhibitor Cocktail, Roche Applied Science). The lysate was centrifuged at 15,000×*g* for 30 min for collection of the supernatant, the concentration of protein in which was determined using a protein assay kit (Bio-Rad, Hercules, CA, USA). Twenty micrograms of protein was mixed with SDS gel-loading buffer and resolved by SDS-polyacrylamide gel electrophoresis on 10–20% gels (Wako Pure Chemical Industries). The proteins were subsequently transferred onto a polyvinylidene fluoride membrane (Millipore, Billerica, MA, USA), which was then blocked for 1 h with Tris-buffered saline-Tween 20 (10 mM Tris-HCl at pH 7.4, 150 mM NaCl, and 0.1% Tween 20) containing 5% skim milk or 5% bovine serum albumin (Sigma, St. Louis, MO, USA) and incubated with antibodies against AMPKα2 (Millipore) or phosphorylated AMPKα (Thr172) (Millipore), respectively. The membranes were also probed with an antibody against α-tubulin (Sigma). Protein bands were visualized with Immobilon Western Detection Reagent (Millipore) and an LAS-4000 mini luminescent image analyzer (Fujifilm, Tokyo, Japan). The relative level of each protein was normalized to that of α-tubulin.

### Statistical analysis

Data are presented as means ± SEM. Statistical analysis comprised repeated-measures one-way ANOVA followed by the Tukey–Kramer test. A *p*-value < 0.05 was considered to indicate a significant difference among means.

## Results

### Body weight and daily ADN and AMP intake

Body weight, body weight gain, and the food efficiency ratio (FER) did not differ among the groups after administration of ADN or AMP for 14 or 25 weeks (Additional file 2: Table S2) when compared with the control group. However, the FER in the ADN group was significantly higher compared with that in the AMP group at 14 weeks. In addition, no differences between the groups were noted in food and water intake and relative organ weights (data not shown). ADN and AMP intake, calculated according to the volume of water consumed, was 25 ± 0.1 and 34 ± 0.3 μg·day^− 1^, respectively.

### Serum parameters

**Fig. 2:**
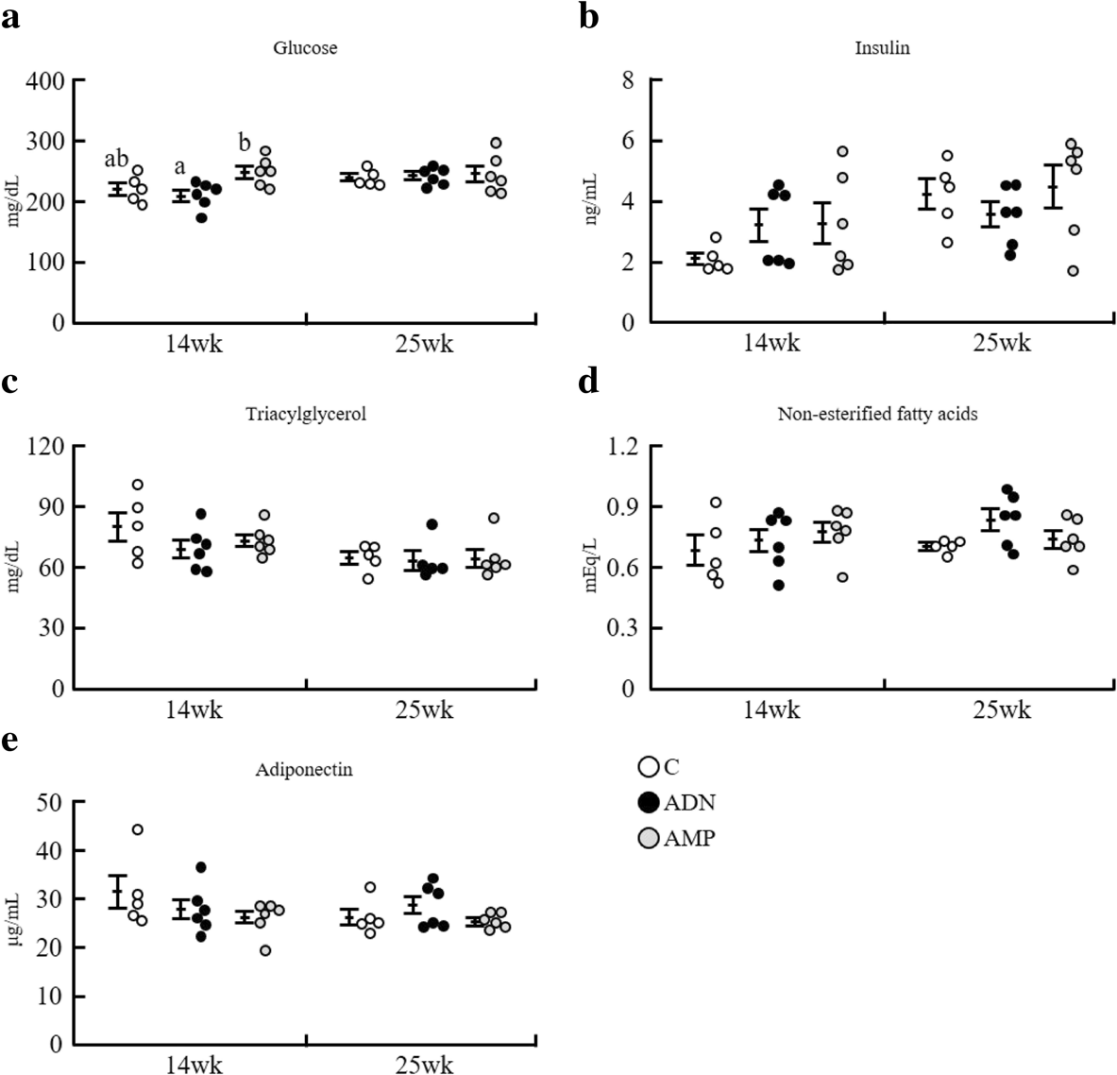
Serum biochemical parameters of mice with high-fat diet-induced obesity administered ADN or AMP after fasting 6 h. **a** Glucose; (**b**) insulin; (**c**) triacylglycerol; (**d**) non-essential fatty acids; (**e**) adiponectin. Values are means ± SEM, *n* = 5 or 6. *p < 0.05 versus the control group. Different letters in the same panel represent a significant difference (*p* < 0.05). C, control group; ADN, adenosine group; AMP, adenosine-5′-monophosphate group

After 6 h of fasting, serum glucose levels at the 14th week of treatment were significantly lower in the ADN group than in the AMP group but did not significantly differ from those in the control group (Fig. 2a). In addition, no differences were noted among the control, ADN, and AMP groups in terms of serum levels of insulin, triacylglycerol, non-esterified fatty acids, and adiponectin (Fig. 2b-e).

### OGTT

**Fig. 3:**
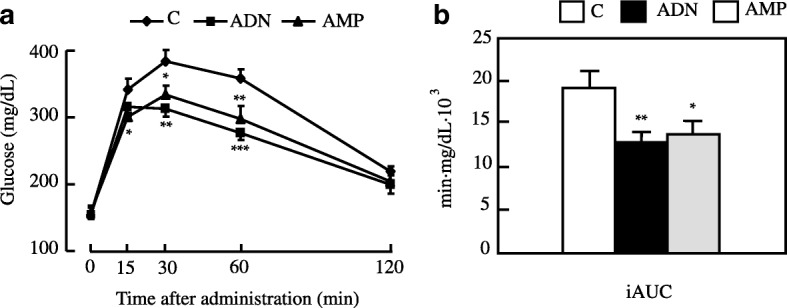
Oral glucose tolerance tests of mice with high-fat diet-induced obesity administered ADN or AMP. **a** Blood glucose changes; (**b**) incremental areas under the curve (iAUC). Values are means ± SEM, n = 5 or 6. *p < 0.05, **p < 0.01, ***p < 0.001 versus the control group. C, control group; ADN, adenosine group; AMP, adenosine-5′-monophosphate group

**Fig. 4:**
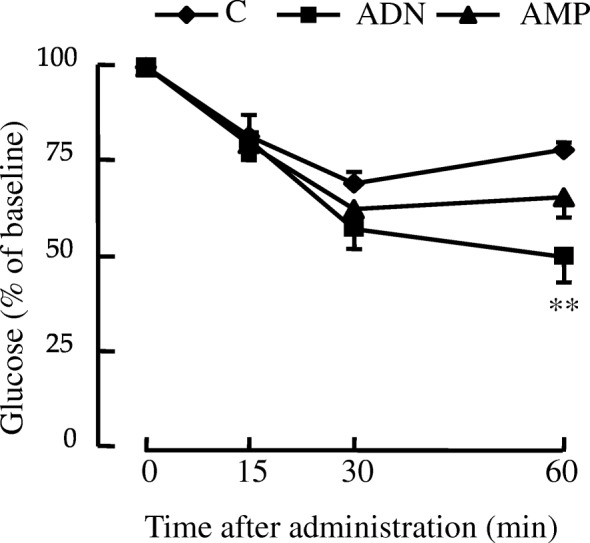
Changes in blood glucose during insulin tolerance tests of mice with high-fat diet-induced obesity administered ADN or AMP. Values are means ± SEM, *n* = 5 or 6. **p < 0.01 versus the control group. C, control group; ADN, adenosine group; AMP, adenosine-5′-monophosphate group

We performed OGTT at the 14th week of administration. Plasma glucose levels in both the ADN and AMP groups were significantly lower than those in the control group 30 and 60 min after glucose administration (Fig. 3a). Furthermore, the incremental area under the curve calculated for the ADN and AMP treatments was significantly smaller than that for the control (Fig. 3b). ITT were carried out at the 15th week of treatment. Plasma glucose levels were significantly lower in the ADN group than in the control group 60 min after administration of insulin (Fig. 4).

### AMPK phosphorylation and gene expression

**Fig. 5:**
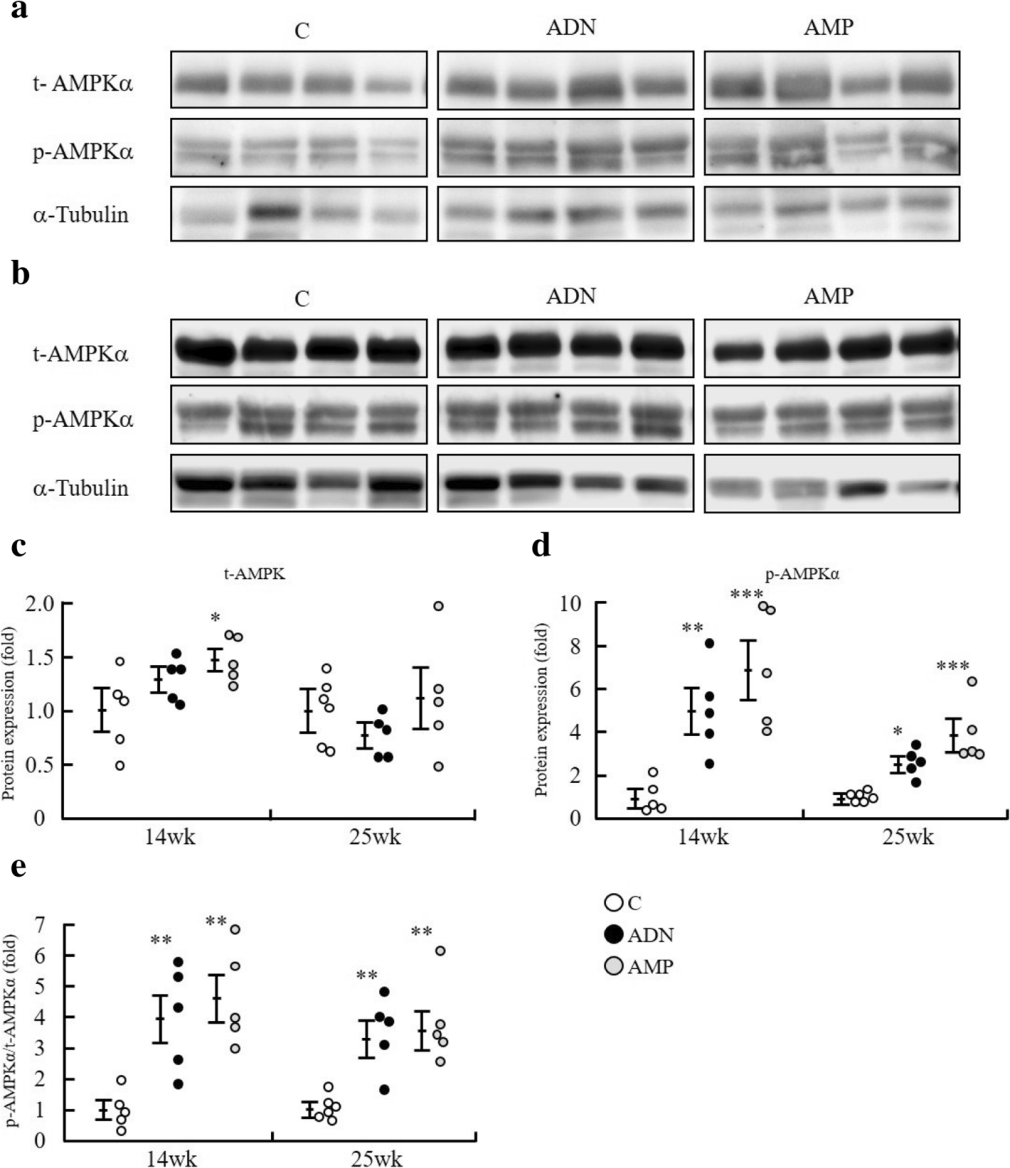
Representative immunoblot images and quantitative analyses of total AMPKα, phosphorylated AMPKα, and α-tubulin. **a** Western blot images at 14 weeks; (**b**) western blot images at 25 weeks; (**c**) expression of total AMPKα (t-AMPKα); (**d**) expression of phosphorylated AMPKα (p-AMPKα); and (**e**) ratio of t-AMPKα to p-AMPKα. Values are means ± SEM, n = 5 or 6. *p < 0.05, **p < 0.01, ***p < 0.001 versus the control group. C, control group; ADN, adenosine group; AMP, adenosine-5′-monophosphate group

We measured levels of phosphorylated and total AMPK α-subunit (AMPKα) protein in skeletal muscle by western blotting to evaluate AMPK activation in this tissue (Fig. 5a, b). The relative quantity of total AMPKα (t-AMPKα) at the 14th week of administration was significantly higher in the AMP group than in the control group (Fig. 5c), and phosphorylated AMPKα (p-AMPKα) levels in the ADN and AMP groups were significantly higher than those in the control group at both week 14 and 25 (Fig. 5d). Moreover, the ratio of p-AMPKα to t-AMPKα was significantly higher in the ADN and AMP groups than in the control group after 14 and 25 weeks of treatment (Fig. 5e). These results suggest that administration of ADN or AMP enhanced AMPK activity in skeletal muscle. mRNA levels of peroxisome proliferator-activated receptor α (*Pparα*) were significantly increased after 14 weeks of ADN or AMP treatment. In addition, those of acyl-CoA synthase (*Acs*), very long chain acyl-CoA dehydrogenase (*Vlcad*), and PPAR gamma coactivator 1α (*Pgc1α*) were significantly higher in the AMP group than in the control group at week 14 (Additional file 3: Table S3). However, no significant differences in the levels of these transcripts were observed between the groups at the 25th week of administration.

## Discussion

In our previous work, we investigated the capacity of ADN and AMP to ameliorate metabolic syndrome-related diseases using a stroke-prone spontaneously hypertensive rat model of non-obesity-associated insulin resistance and hypertension-related disorders similar to human essential hypertension [20, 21]. ADN administration reduced blood glucose and insulin levels measured under fasting conditions, as well as glucose and insulin tolerance. In addition, we showed for the first time that ingestion of AMP increases plasma adiponectin concentration, upregulates hepatic *Prkaa1* mRNA expression, and elevates levels of p-AMPKα, leading to enhanced expression of genes associated with β-oxidation in the liver.

In the current study, we examined the effect of ADN and AMP administration on glucose metabolism using mice fed a high-fat diet as an animal model of obesity. Human and animal studies have demonstrated that a diet high in fat induces increased lipid accumulation in tissues including adipose tissue and skeletal muscle, followed by insulin resistance [24]. Increased levels of non-esterified fatty acids in the blood and diacylglycerol and palmitoyl-CoA in organs result in activation of c-Jun N-terminal kinase (JNK) and inhibitor of kappa light polypeptide gene enhancer in B-cells, kinase β (IKKβ). Activation of JUN and IKKβ leads to serine phosphorylation of insulin receptor substrate, interfering with insulin signaling via the insulin receptor [25, 26]. We speculate that the effect of ADN and AMP is more pronounced in the early stage of diabetes. Future studies are needed to clarify the detailed mechanism, but ADN and AMP are promising candidates for the prevention of diabetes in both mice and humans.

Even though blood glucose levels in fasted state were not different among the groups (Fig. 1a), tolerance tests revealed that ADN and AMP improved the abnormal glucose tolerance (Fig. 3) and insulin sensitivity (Fig. 4) observed in the mouse model of obesity used in this study. These results indicate that administration of ADN or AMP can mitigate insulin resistance in mice fed a high-fat diet. ADN and AMP treatment may have the ability to reduce blood glucose in postprandial, but not in fasted state.

In this study, we found that oral administration of ADN or AMP can activate AMPK in skeletal muscle (Fig. 5). This is the first in vivo demonstration of AMPK activation by ADN or AMP in the high-fat diet-induced obese mouse model of insulin resistance. AMPK acts as a sensor, and when activated, stimulates mitochondrial biogenesis, energy production, lipid oxidation, and glucose influx, alleviating insulin resistance via activation of sirtuin 1 and PGC1α [27]. AMPK activation in muscle was enhanced after both 14 and 25 weeks of ADN or AMP administration independently of adiponectin. Thus, ADN and AMP may indeed suppress the progression of high-fat diet-induced insulin resistance relatively early in the development of diabetes.

mRNA expression of the gene encoding PPARα, an AMPK target in skeletal muscle, was increased in the AMP and ADN groups after 14 weeks of administration. Furthermore, levels of *Acs* and *Vlcad* mRNA were also higher in the AMP group at this time point (Additional file 3: Table S3). However, these differences were no longer evident after 25 weeks of treatment, despite continued AMPK activation in the skeletal muscle of ADN- or AMP-treated mice. Thus, although at 14 weeks (Additional file 3: Table S3), activation of AMPK in skeletal muscle stimulated the expression of PPARα and its target genes; it suggests that an adaption had occurred by the 25th week of administration (Additional file 3: Table S3). Skeletal muscle can adapt its metabolic properties in response to a number of physiologic conditions by activation and repression of signaling events that affect the metabolic pathways of several genes related to the oxidation process. The first evidence linking AMPK to the regulation of glucose metabolism in skeletal muscle was provided by treatment with AICAR, an activator of AMPK that enhances fatty acid oxidation [28]. Our results suggest that ADN and AMP contribute to improving the regulation of glucose metabolism through expression of PPARα and its target genes.

Lipid accumulation in the skeletal muscle of obese animals induces abnormal glucose metabolism accompanied by dysregulated insulin signaling and skeletal muscle function. Upregulation of lipid oxidation in skeletal muscle is considered a therapeutic strategy to combat insulin resistance. Unsaturated fatty acids, such as oleic acid, and agonists of PPARα enhance lipid oxidation and are expected to have therapeutic effects in diabetes [29–31]. In our study, administration of ADN or AMP increased the expression of transcripts associated with lipid oxidation in skeletal muscle. As muscle is the major site of ATP production and consumption, enhanced lipid oxidation in skeletal muscle may be involved in the mitigation of abnormal glucose metabolism by ADN and AMP in mice fed a high-fat diet.

Although this study was carefully and well prepared, we were still aware of its limitations. We need to measure locomotor activity of mice. Various studies have shown that in rodent models of obesity locomotor activity is necessary to evaluate. Further studies are needed to evaluate the behavior of mice after administration of ADN and AMP. In addition, further studies are needed to elucidate the complete physiological effect of ADN and AMP to confirm its detailed mechanism of action at the molecular level.

## Conclusions

Here, we showed that administration of ADN or AMP to obese mice ameliorates abnormal glucose metabolism induced by a high-fat diet. ADN or AMP treatment also increases the level of activated AMPK in skeletal muscle. Activation of AMPK may upregulate lipid oxidation and attenuates insulin resistance caused by obesity.

## Additional files

Additional file 1: **Table S1.** Sequences of primers used for quantitative RT-PCR. (DOCX 18 kb)

Additional file 2: **Table S2.** Effect of ADN and AMP on body weight, weight gain, and food efficiency ratio. (DOCX 13 kb)

Additional file 3: **Table S3.** Quantitative RT-PCR measurements of mRNA levels (fold changes) after ADN and AMP administration. (DOCX 17 kb)

## Abbreviations

ADN: Adenosine
AMP: Adenosine-5′-monophosphate
AMPK: AMP-activated protein kinase
ITT: Insulin tolerance tests
OGTT: Oral glucose tolerance tests
