# Supplementary material for: Adenosine and adenosine-5′-monophosphate ingestion ameliorates abnormal glucose metabolism in mice fed a high-fat diet
Source: BMC Complement Altern Med. 2018 Nov 14;18:304. doi: 10.1186/s12906-018-2367-6 (PMC6236947; doi:10.1186/s12906-018-2367-6)
Supplement: Supplementary file 1 — Table S1. Sequences of primers used for quantitative RT-PCR. (DOCX 18 kb) [file 12906_2018_2367_MOESM1_ESM.docx]

Table S1. Sequences of primers used for quantitative RT-PCR

| Gene | Forward primer | Reverse primer |
| --- | --- | --- |
| *Eef-1* | GATGGCCCCAAATTCTTGAAG | GGACCATGTCAACAATTGCAG |
| *Pparα* | AATTTGCTGTGGAGATCGGC | TCCTGCAACTTCTCAATGTAGCC |
| *Acs* | GGACGACTTGTTGAAACTTGGG | CTTTGACCTGTTCAAATGGCTTC |
| *Vlcad* | GGCGGTTGATCATGCTACTAATC | CATGTTGGCACTCAGCATGTAAG |
| *Lcad* | GCTTCCACAGGAAAGGCTCTTAA | TATGCTGCACCGTCTGTATGTGT |
| *Mcad* | TTGGCACGTTCTAACCCAGATC | CTGGCCCATGTTTAGTTCCTTT |
| *Acox* | TGATGAAATATGCCCAGGTGAA | CACTGTATCGAATGGCAATGGT |
| *Cpt2* | GGTCGATGAAAAGCCTCCATT | CTTTCCAACCCGATCTCCTTAA |
| *Pgc1α* | CCGGAGTATGACACCGTATTTG | TCTGCTCTCACGTCTGAAGTTCTT |
| *Glut4* | GCTTTGTGGCCTTCTTTGAGA | CCCATAGCATCCGCAACATACT |

*Eef-1*, eukaryotic elongation factor-1α1; *Pparα*, peroxisome proliferator-activated receptor α; *Acs*, acyl-CoA synthase; *Vlcad*, very long chain acyl-CoA dehydrogenase; *Lcad*, long chain acyl-CoA dehydrogenase; *Mcad*, medium chain acyl-CoA dehydrogenase; *Acox*, acyl-CoA oxidase; *Cpt2*, carnitine palmitoyltransferase 2; *Pgc1α*, peroxisome proliferator-activated receptor gamma, coactivator 1α; *Glut4*, glucose transporter type 4.
