# Supplementary material for: Adenosine and adenosine-5′-monophosphate ingestion ameliorates abnormal glucose metabolism in mice fed a high-fat diet
Source: BMC Complement Altern Med. 2018 Nov 14;18:304. doi: 10.1186/s12906-018-2367-6 (PMC6236947; doi:10.1186/s12906-018-2367-6)
Supplement: Supplementary file 2 — Table S2. Effect of ADN and AMP on body weight, weight gain, and food efficiency ratio. (DOCX 13 kb) [file 12906_2018_2367_MOESM2_ESM.docx]

Table S2. Effect of ADN and AMP on body weight, weight gain, and food efficiency ratio

|  | 14 weeks | | | 25 weeks | | |
| --- | --- | --- | --- | --- | --- | --- |
|  | C | ADN | AMP | C | ADN | AMP |
| Body weight (g) | 38.2±0.49 | 39.9±1.07 | 40.0±1.61 | 48.0±0.69 | 46.8±0.68 | 45.0±1.81 |
| Weight gain (g) | 19.3±0.47 | 20.9±0.77 | 20.8±1.59 | 27.0±0.69 | 25.5±0.72 | 23.8±1.87 |
| FER^#^ | 0.081±0.002 | 0.088±0.003 | 0.074±0.005^*^ | 0.063±0.002 | 0.062±0.002 | 0.059±0.004 |

Values are means ± SEM, n = 5 or 6. C, control group; ADN, adenosine group; AMP, adenosine-5′-monophosphate group. *(P<0.05), significant difference when compared with the control group (14 weeks). ^#^Food efficiency ratio (FER) = weight gain (g) / total food intake (g).
