# Supplementary material for: Adenosine and adenosine-5′-monophosphate ingestion ameliorates abnormal glucose metabolism in mice fed a high-fat diet
Source: BMC Complement Altern Med. 2018 Nov 14;18:304. doi: 10.1186/s12906-018-2367-6 (PMC6236947; doi:10.1186/s12906-018-2367-6)
Supplement: Supplementary file 3 — Table S3. Quantitative RT-PCR measurements of mRNA levels (fold changes) after ADN and AMP administration. (DOCX 17 kb) [file 12906_2018_2367_MOESM3_ESM.docx]

Table S3. Quantitative RT-PCR measurements of mRNA levels (fold changes) after ADN and AMP administration

|  | 14 weeks | | | 25 weeks | | |
| --- | --- | --- | --- | --- | --- | --- |
| Gene | C | ADN | AMP | C | ADN | AMP |
| *Pparα* | 1.0±0.2 | 1.4±0.1^*^ | 1.6±0.2^*^ | 1.0±0.2 | 1.2±0.2 | 1.2±0.2 |
| *Acs* | 1.0±0.1 | 1.1±0.1 | 1.3±0.1^*^ | 1.0±0.1 | 1.0±0.1 | 1.0±0.2 |
| *Vlcad* | 1.0±0.1 | 1.2±0.1 | 1.4±0.1^*^ | 1.0±0.2 | 1.0±0.1 | 0.9±0.1 |
| *Lcad* | 1.0±0.1 | 1.4±0.1 | 1.2±0.1 | 1.0±0.1 | 1.1±0.1 | 0.9±0.1 |
| *Mcad* | 1.0±0.1 | 1.2±0.1 | 1.3±0.1 | 1.0±0.2 | 1.0±0.1 | 1.0±0.1 |
| *Acox* | 1.0±0.1 | 1.1±0.1 | 1.2±0.1 | 1.0±0.1 | 1.0±0.1 | 0.9±0.1 |
| *Cpt2* | 1.0±0.1 | 1.2±0.1 | 1.3±0.1 | 1.0±0.1 | 1.0±0.1 | 0.9±0.1 |
| *Pgc1α* | 1.0±0.1 | 1.1±0.1 | 1.4±0.1^*^ | 1.0±0.1 | 0.9±0.1 | 0.9±0.1 |
| *Glut4* | 1.0±0.1 | 1.1±0.2 | 1.3±0.1 | 1.0±0.1 | 1.0±0.2 | 1.1±0.2 |

Values are means ± SEM, n = 5 or 6. C, control group; ADN, adenosine group; AMP, adenosine-5′-monophosphate group. *(P<0.05), significant difference when compared with the control group (14 weeks). *Pparα*, peroxisome proliferator-activated receptor α; *Acs*, acyl-CoA synthase; *Vlcad*, very long chain acyl-CoA dehydrogenase; *Lcad*, long chain acyl-CoA dehydrogenase; *Mcad*, medium chain acyl-CoA dehydrogenase; *Acox*, acyl-CoA oxidase; *Cpt2*, carnitine palmitoyltransferase 2; *Pgc1α*, peroxisome proliferator-activated receptor gamma, coactivator 1α; *Glut4*, glucose transporter type 4.
